# Identifying druggable gene-related biomarkers in intervertebral disc degeneration through transcriptome sequencing and mendelian randomization analysis

**DOI:** 10.3389/fgene.2026.1627091

**Published:** 2026-01-23

**Authors:** Keping Wang, Zuolong Wu, Guanghai Zhao, Shaolong Li, Yizhe Lou, Guanhong Pan, Guangyi Wang, Haihong Zhang

**Affiliations:** 1 Orthopedics Department, Lanzhou University Second Hospital, Lanzhou, Gansu, China; 2 The Second Clinical Medical College, Lanzhou University, Lanzhou, Gansu, China

**Keywords:** diagnostic model, intervertebral disc degeneration, mendelian randomization, neutrophils, potential druggable genes

## Abstract

**Background:**

Intervertebral disc degeneration (IDD) is a major contributor to low back pain, yet its molecular mechanisms remain unclear. Identifying potential druggable genes (PDGs) associated with IDD could facilitate early diagnosis and targeted therapy. This study aimed to explore the diagnostic and mechanistic significance of PDGs in IDD.

**Methods and Results:**

Three GEO datasets were merged as a training set, with another blood-based dataset as testing. PDGs were obtained from the literature and intersected with differentially expressed genes (DEGs). Functional enrichment and immune infiltration analyses were performed. A Lasso regression model was developed for diagnosis, and Mendelian Randomization (MR) analysis inferred causality. Cellular experiments validated key gene expression. Fourteen differentially expressed PDGs were identified, primarily involved in immune responses and neutrophil activity. A five-gene diagnostic model (BPI, CD160, CTSG, CYP27A1, KIF11) was constructed and demonstrated high accuracy. MR analysis confirmed a causal relationship between BPI and CTSG with IDD. GSEA revealed that BPI was negatively associated with oxidative phosphorylation, while CTSG was linked to the G2M checkpoint. Cellular experiments confirmed BPI and CTSG upregulation in TNF-α-induced NPCs.

**Conclusion:**

This study constructed a diagnostic model and identified BPI and CTSG as potential biomarkers for IDD, providing new insights into IDD treatment.

## 1 Introduction

Low back pain (LBP) is one of the leading causes of disability worldwide (Xin et al., 2022). It is estimated that 70%–85% of individuals will experience LBP at some point in their lifetime, significantly limiting their mobility and imposing a substantial socioeconomic burden (Samanta et al., 2023). Although the exact pathogenesis of LBP remains unclear, intervertebral disc degeneration (IDD) is widely recognized as a primary cause (Xiang et al., 2023). Each intervertebral disc consists of the central nucleus pulposus (NP), the outer annulus fibrosus (AF), and the superior/inferior cartilage endplates (Chen et al., 2023). IDD is characterized by pathological changes such as extracellular matrix (ECM) degradation, increased inflammatory responses, elevated oxidative stress levels, apoptosis, and cellular senescence (Li et al., 2022). Its underlying mechanisms involve multiple factors, including genetic susceptibility, abnormal mechanical loading, aging, and lifestyle influences (Cazzanelli and Wuertz-Kozak, 2020). Currently, conventional treatments for IDD include surgical intervention, pharmacotherapy, and physical therapy; however, these approaches mainly alleviate symptoms and fail to reverse or halt the degenerative process (Shi et al., 2024). Therefore, identifying novel therapeutic targets and early diagnostic biomarkers is crucial for improving IDD management and intervention strategies.

Identifying suitable drug targets is a critical step in drug development, and the concept of the “druggable genome” has significantly facilitated the discovery of repurposable drug targets (Yin et al., 2024). Potentially druggable genes (PDGs) are a class of genes encoding proteins whose activity can be modulated by small-molecule drugs, peptides, or biologics, making them potential therapeutic targets (Wang Li et al., 2024). In recent years, Mendelian randomization (MR) analysis, which integrates genome-wide association studies (GWAS) and expression quantitative trait loci (eQTL) data, has been widely used to identify novel therapeutic targets (Liu et al., 2024). To date, studies have explored PDG-based therapeutic targets in several diseases, including Parkinson’s disease (Lyu et al., 2024), post-ischemic stroke (Zhang et al., 2024), lung cancer (Song et al., 2024), and diabetes (Li et al., 2024). Since the introduction of the druggable genome concept, more than 6,000 genes have been identified as part of the PDG, with their activity potentially modifiable by drugs (Jiang et al., 2022). There were < 10% of these PDGs that have been approved as drug targets by the Food and Drug Administration (FDA) (Santos et al., 2017). To our knowledge, no study has systematically investigated the role of PDGs in the pathogenesis of IDD.

This study aims to identify key biomarkers associated with PDGs IDD by integrating transcriptomic data with MR analysis, thereby uncovering potential druggable targets and their regulatory mechanisms. The findings of this study will provide a solid theoretical foundation for the precise treatment of IDD and offer new insights into biomarker screening and targeted therapeutic strategies, ultimately enhancing the clinical management and treatment outcomes for IDD patients.

## 2 Methods

### 2.1 Data source and preprocessing

The expression profile datasets of IDD tissue samples, GSE34095, GSE124272, and GSE147383, were downloaded from the GEO database (https://www.ncbi.nlm.nih.gov/geo/). The removeBatchEffect function from the limma package was used to merge these three datasets and eliminate batch effects. The merged dataset included 15 disease tissue samples and 15 normal control tissue samples. Additionally, the expression profile datasets GSE150408 (17 normal and disease samples) and GSE70362 (16 normal and 32 disease samples) were also downloaded as external validation.

### 2.2 Identification of differentially expressed PDGs

The limma package was used to analyze the differential gene expression between the disease group and the normal group in both the integrated dataset and GSE150408. The screening criteria were set as |log2FC| > 0.5 and p < 0.05. Based on previously published literature, a set of 4,463 PDGs was identified (Finan Kruger et al., 2017). The VennDiagram package was used to generate a Venn diagram to identify druggable genes that were differentially expressed in both datasets.

### 2.3 Functional enrichment analysis

To explore the biological functions of the identified druggable genes, GO and KEGG pathway enrichment analyses were conducted using the clusterProfiler package. The significance threshold was set at p < 0.05. The results were visualized using the ggplot2 package.

### 2.4 Immune cell infiltration analysis

The CIBERSORT algorithm was employed to predict the distribution of immune cells in the samples, and a t-test was conducted to compare the proportions of immune cells between the disease and normal groups, aiming to investigate immune cell infiltration changes between the two groups. Furthermore, to ensure the reliability of the results, the MCPcounter and TIMER algorithms were used for additional validation.

### 2.5 Construction of diagnostic model

To further explore the diagnostic role of the differentially expressed druggable genes in IDD, Lasso regression analysis was performed on the integrated dataset using the glmnet package. A 5-fold cross-validation was conducted, with the lambda parameter set to 0.05784, to obtain the optimal binary classification diagnostic model. Additionally, to prevent overfitting, the classification performance of the model was also validated in GSE150408. The pROC package was used to generate ROC curves to quantify the predictive performance of the model.

### 2.6 MR analysis

The GWAS data for the exposure factors (diagnostic model characteristic genes) and outcome variables were obtained from the IEU Open GWAS Project (https://gwas.mrcieu.ac.uk/), and the details are shown in Supplementary Table S1. The extract_instruments function in the TwoSampleMR package was used to select SNPs that were strongly associated with the exposure factors from the GWAS data, using a threshold of *P*-value < 5e-08. The clump_data function was then applied to remove SNPs with linkage disequilibrium to ensure the independence of instrumental variables, with the parameters set as clump_r2 = 0.001 and clump_kb = 10,000. MR analysis was performed using the inverse variance weighted (IVW) method, weighted median (WM) estimation, and MR-Egger regression, implemented in the TwoSampleMR package. Additionally, IVW and MR-Egger tests were conducted to assess the presence of heterogeneity and pleiotropy in the results.

### 2.7 Cell culture and treatment

Human NP cells (NPCs) were purchased from ScienCell (CA, USA) and cultured in Dulbecco’s Modified Eagle Medium (DMEM) supplemented with 10% fetal bovine serum (FBS) and 1% penicillin-streptomycin in T25 culture flasks at 37 °C in a humidified incubator with 5% CO_2_. When NPCs reached 90% confluence, they were detached using 0.25% trypsin/1 mM EDTA and subcultured into larger flasks for expansion. Third- and fourth-generation NPCs were used for subsequent experiments.

For TNF-α treatment, NPCs were seeded at a density of 5,000 cells per well in 96-well plates with 200 μL of complete culture medium and incubated overnight at 37 °C with 5% CO_2_. The next day, the medium was removed, and cells were washed with PBS. Fresh medium containing TNF-α (100 ng/mL) was then added for 24 h (Duan Kuang et al., 2023). Cells were incubated under these conditions for the indicated time points before further analysis.

### 2.8 Cell counting kit 8 (CCK-8) analysis

Briefly, NPCs were seeded in 96-well plates at a density of 5,000 cells per well and incubated at 37 °C with 5% CO_2_ overnight. After the designated treatments, 10 μL of CCK-8 reagent was added to each well, followed by incubation at 37 °C for 2 h. The absorbance at 450 nm was measured using a microplate reader.

### 2.9 Reverse transcription-quantitative polymerase chain reaction (RT-qPCR)

Total RNA was extracted from NPCs using TRIzol reagent (Invitrogen, CA, USA). The RNA concentration and purity were assessed using a NanoDrop spectrophotometer (Thermo Fisher Scientific, CA, USA). For cDNA synthesis, 1 μg of total RNA was reverse-transcribed using the PrimeScript RT reagent Kit following the manufacturer’s protocol. The reaction was carried out at 37 °C for 15 min, followed by 85 °C for 5 s to inactivate the reverse transcriptase. RT-qPCR was performed using TB Green™ Premix Ex Taq™ on a QuantStudio™ 5 Real-Time PCR System. The thermal cycling conditions were as follows: 95 °C for 30 s, followed by 40 cycles of 95 °C for 5 s and 60 °C for 30 s. Gene expression levels were normalized to GAPDH as an internal control, and relative expression was calculated using the 2^−ΔΔCT^ method. Each experiment was performed in triplicate to ensure reproducibility.

### 2.10 Flow cytometry for cell apoptosis

NPCs were harvested and washed twice with cold PBS, then resuspended in 100 μL of binding buffer. Apoptosis was assessed using the Annexin V-FITC/PI Apoptosis Detection Kit following the manufacturer’s protocol. Briefly, cells were stained with 5 μL of Annexin V-FITC and 5 μL of propidium iodide (PI) and incubated in the dark at room temperature for 15 min. After staining, 400 μL of binding buffer was added to each sample, and apoptosis was analyzed using a flow cytometer within 1 h. The proportion of apoptotic cells was determined using FlowJo software.

### 2.11 Western blotting

NPCs were lysed using RIPA buffer on ice for 30 min. The lysates were centrifuged at 12,000 *× g* for 15 min at 4 °C, and the supernatants were collected. Protein concentration was determined using the BCA protein assay kit. Equal amounts of protein were mixed with 5× loading buffer, boiled at 95 °C for 5 min, and separated on a 10% or 12% SDS-PAGE gel. A pre-stained protein molecular weight marker (#G2086-250UL, Servicebio, Hubei, China) was loaded in parallel. The proteins were then transferred onto a PVDF membrane using a wet transfer system at 300 mA for 90 min. After blocking with 5% non-fat milk or 5% BSA in TBST for 1 h at room temperature, the membranes were incubated overnight at 4 °C with primary antibodies against BPI (1:1,000, #ab175231, Abcam, UK), CTSG (1:1,000, #ab282105, Abcam, UK), MMP-13 (1:1,000, #ab39012, Abcam, UK), Collagen II (1:1,000, #ab188570, Abcam, UK), and GAPDH (1:10,000, #ab128915, Abcam, UK) as a loading control. The next day, membranes were washed with TBST and incubated with HRP-conjugated secondary antibodies (1:2,000, #ab288151, Abcam, UK) for 1 h at room temperature. Protein bands were visualized using an ECL detection reagent. The relative expression levels of BPI and CTSG were quantified using ImageJ software.

### 2.12 Enzyme-linked immunosorbent assay (ELISA)

The concentrations of inflammatory cytokines IL-6 and COX-2 in the culture supernatants of NPCs were determined using ELISA kits according to the manufacturer’s instructions. Briefly, after siRNA transfection and TNF-α treatment, cell culture media were collected and centrifuged at 1,000 × g for 10 min at 4 °C to remove debris. The supernatants were then subjected to ELISA analysis using human IL-6 and COX-2 ELISA kits. Optical density values were measured at 450 nm using a microplate reader, and cytokine concentrations were calculated based on standard curves generated from serial dilutions of known standards.

### 2.13 Statistical analysis

All statistical analyses were performed using R software (version 4.3.2) and GraphPad Prism (version 9.0). Data are presented as the mean ± standard deviation. Differences between two groups were assessed using the unpaired Student’s t-test. Pearson correlation analysis was used to assess relationships between variables. Statistical significance was defined as p < 0.05.

## 3 Results

### 3.1 Identification of differentially expressed PDGs in IDD

**FIGURE 1:**
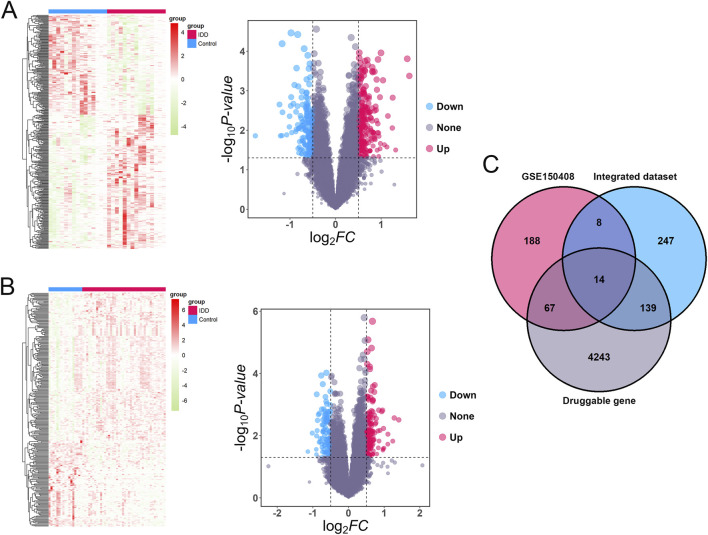
Identification of differentially expressed PDGs in IDD. **(A)** Heatmap and volcano plot of EDGs in integrated dataset. **(B)** Heatmap and volcano plot of EDGs in GSE150408 dataset. **(C)** Venn diagram revealed 14 differentially expressed PDGs in IDD.

First, three GEO datasets (GSE34095, GSE124272, and GSE147383) were merged as the training set. After removing batch effects, a uniform sample distribution was observed. A total of 408 DEGs were identified from the merged dataset, including 174 downregulated and 234 upregulated genes (Figure 1A). In the validation set GSE150408, 277 DEGs were obtained (102 downregulated and 175 upregulated, Figure 1B). The intersection of these DEGs with 4,463 PDGs yielded 14 overlapping genes (Figure 1C), identified as differentially expressed PDGs in IDD. The detailed gene names are listed in Table 1.

**TABLE 1:** Differentially expressed druggable genes in IDD.

Number	Genes	Number	Genes
1	CYP27A1	8	CYP4F2
2	AZU1	9	CD160
3	FCGR1A	10	CTSG
4	FGFBP1	11	CEACAM8
5	FCGBP	12	DEFA4
6	MPO	13	BPI
7	KIF11	14	CRISPLD2

### 3.2 Functions of differentially expressed PDGs in IDD

**FIGURE 2:**
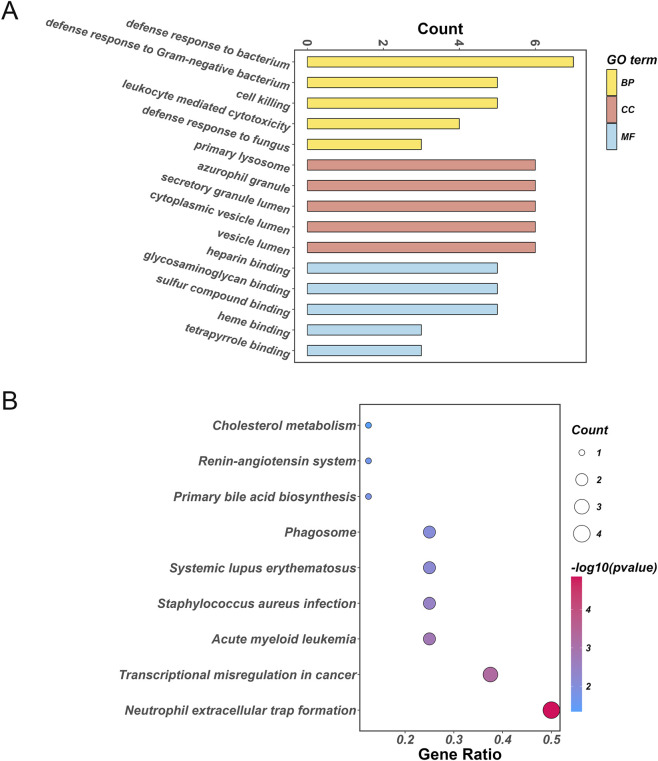
Functions of differentially expressed PDGs in IDD. **(A)** GO enrichment results. **(B)** KEGG enriched pathways.

To investigate the functional roles of these 14 PDGs, GO and KEGG enrichment analyses were performed (Figure 2). GO analysis revealed that biological processes (BP) were primarily enriched in defense responses to bacteria (especially Gram-negative bacteria) and fungi, cell killing, and leukocyte-mediated cytotoxicity (Figure 2A). The top 5 cellular components (CC) included primary lysosome, azurophil granule, secretory granule lumen, and cytoplasmic vesicle lumen (Figure 2A). Molecular functions (MF) were enriched in heparin binding, glycosaminoglycan binding, sulfur compound binding, heme binding, and tetrapyrrole binding (Figure 2A). Key KEGG pathways included neutrophil extracellular trap formation, acute myeloid leukemia, and *Staphylococcus aureus* infection (Figure 2B).

### 3.3 Immune cell infiltration revealed the importance of neutrophils in IDD and its correlation with differentially expressed PDGs

**FIGURE 3:**
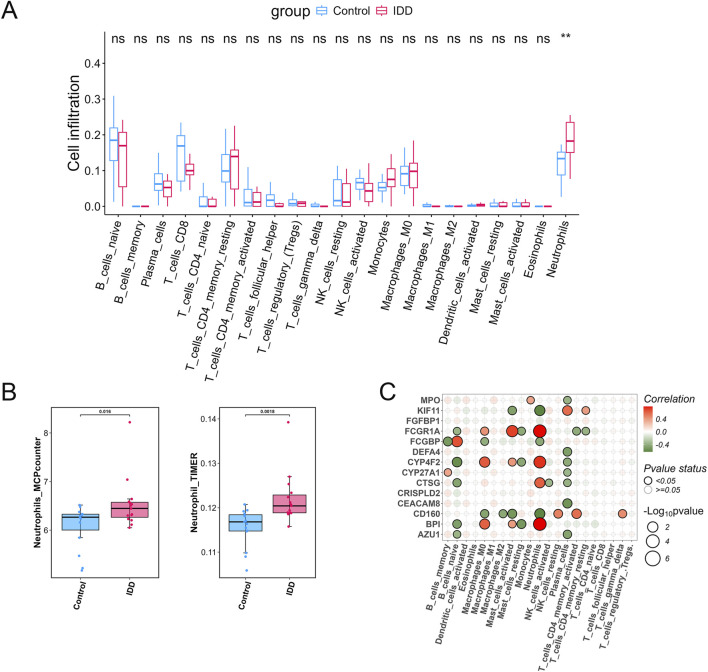
Immune cell infiltration revealed the importance of neutrophils in IDD and its correlation with differentially expressed PDGs. **(A)** Immune infiltration analysis using CIBERSORT. **(B)** Immune infiltration analysis using MCPcouter and TIMER. **(C)** Correlation of 14 PDGs with immune cells in CIBERSORT. ns: no significant, **p < 0.01.

Given the association of these 14 genes with antimicrobial immune responses, immune infiltration analysis was conducted. The CIBERSORT algorithm revealed that only neutrophil infiltration levels showed significant differences between IDD and normal samples, with an upregulation observed in IDD (p < 0.01, Figure 3A). To verify the stability of these findings, immune infiltration analysis was further conducted using two additional algorithms. The results consistently demonstrated significantly higher neutrophil levels in IDD samples compared to normal samples (p < 0.05, Figure 3B). A correlation heatmap was used to explore the relationships between the 14 PDGs and immune cells. Notably, neutrophils exhibited significant negative correlations with KIF11, FCGBP, and CD160, while showing positive correlations with FCGR1A, CYP4F2, CTSG, and BPI (Figure 3C).

### 3.4 Evaluation and validation of diagnostic model

**FIGURE 4:**
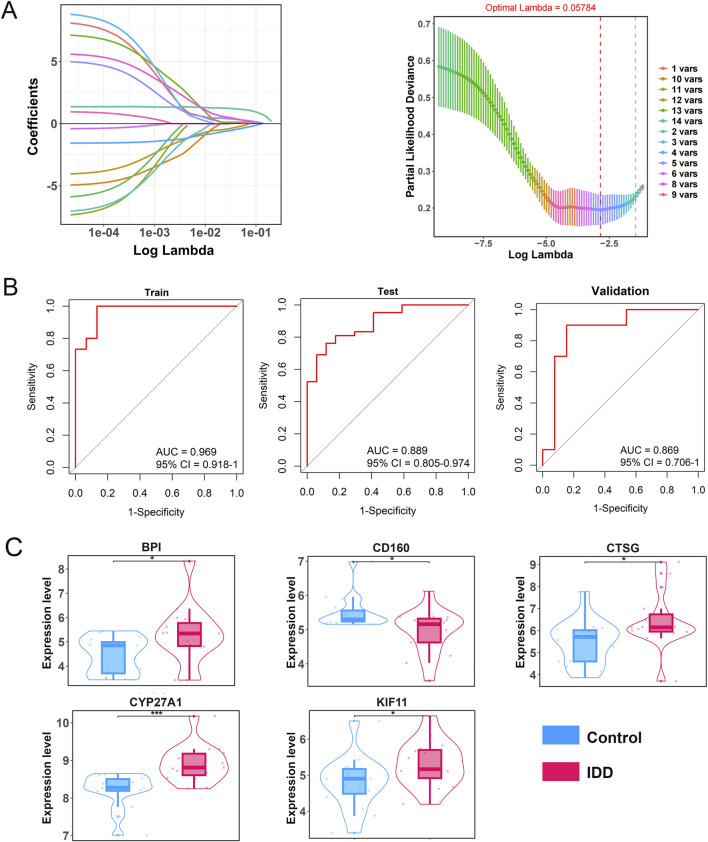
Evaluation and validation of diagnostic model. **(A)** Lasso regression analysis. **(B,C)** ROC curve revealed the diagnostic value of the model in the training (Merge dataset), testing (GSE150408), and validation (GSE70362) datasets. **(C)** Expression of 5 model genes in control and IDD samples. *p < 0.05, **p < 0.001.

To explore the clinical diagnostic value of these 14 PDGs in IDD, we constructed a 5-gene diagnostic model using Lasso regression (Figure 4A), comprising BPI, CD160, CTSG, CYP27A1, and KIF11. This model demonstrated strong diagnostic performance in the training, testing, and validation sets, with AUC values of 0.969, 0.889, and 0.869, respectively (Figure 4B). Next, we examined the expression levels of the five model genes in normal and IDD samples. The results showed that BPI, CTSG, CYP27A1, and KIF11 were significantly upregulated in IDD, while CD160 was significantly downregulated (p < 0.05, Figure 4C).

### 3.5 MR analysis revealed causal relationship between BPI/CTSG and IDD

**FIGURE 5:**
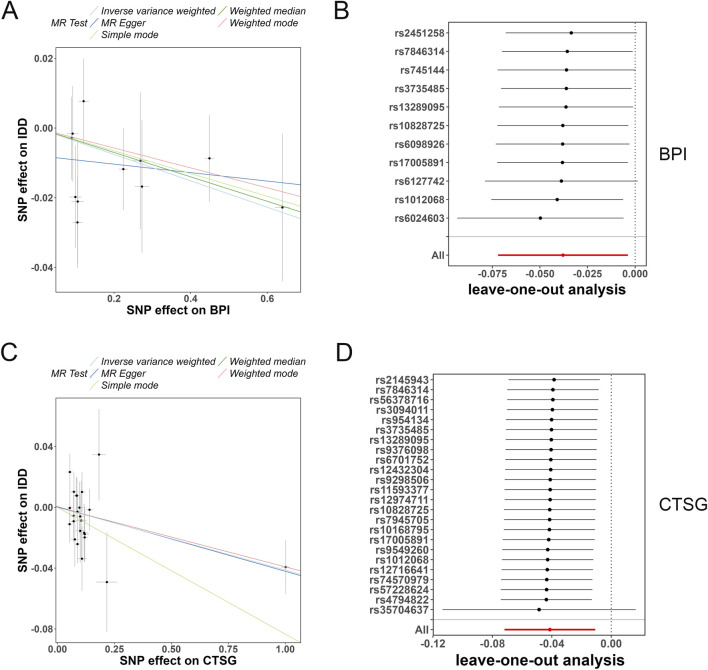
MR analysis revealed causal relationship between BPI/CTSG and IDD. **(A,B)** SNP effect plot and leave-one-out analysis between IDD and BPI. **(C,D)** SNP effect plot and leave-one-out analysis between IDD and CTSG.

Subsequently, we further explored the genetic association between the five model genes and IDD through MR analysis. Among the five MR methods applied, only BPI and CTSG exhibited significant causal associations with IDD (Table 2). SNP effect plots indicated a negative directional association between both BPI and CTSG and IDD (Figures 5A,C). Cochran’s Q test and MR-Egger intercept suggested that the heterogeneity and pleiotropy in the IDD-BPI and IDD-CTSG associations were negligible (all p > 0.05, Tables 3, 4). Additionally, leave-one-out analysis confirmed the robustness of the findings, as all effect estimates remained on the same side (Figures 5B,D).

**TABLE 2:** MR analysis between five model genes with IDD.

Symbol	Exposure	Outcome	Method	nSNP	OR (95%CI)
BPI	eqtl-a-ENSG00000101425	IDD	MR Egger	11	0.988 (0.933–1.046)
			Weighted median	11	0.966 (0.926–1.007)
			Inverse variance weighted	11	0.963 (0.931–0.996)*
			Simple mode	11	0.968 (0.915–1.024)
			Weighted mode	11	0.972 (0.931–1.015)
CD160	eqtl-a-ENSG00000117281	IDD	MR Egger	6	0.985 (0.740–1.312)
			Weighted median	6	0.962 (0.851–1.087)
			Inverse variance weighted	6	0.952 (0.848–1.068)
			Simple mode	6	0.957 (0.771–1.188)
			Weighted mode	6	0.962 (0.844–1.095)
CTSG	eqtl-a-ENSG00000100448	IDD	MR Egger	24	0.958 (0.923–0.995)*
			Weighted median	24	0.962 (0.930–0.995)*
			Inverse variance weighted	24	0.960 (0.931–0.989)**
			Simple mode	24	0.920 (0.805–1.051)
			Weighted mode	24	0.962 (0.930–0.995)*
CYP27A1	eqtl-a-ENSG00000135929	IDD	MR Egger	21	1.034 (0.995–1.074)
			Weighted median	21	1.013 (0.982–1.046)
			Inverse variance weighted	21	1.011 (0.983–1.040)
			Simple mode	21	0.955 (0.871–1.046)
			Weighted mode	21	1.016 (0.984–1.049)
KIF11	eqtl-a-ENSG00000138160	IDD	MR Egger	11	0.894 (0.635–1.259)
			Weighted median	11	1.002 (0.839–1.196)
			Inverse variance weighted	11	1.075 (0.949–1.218)
			Simple mode	11	1.011 (0.765–1.335)
			Weighted mode	11	0.999 (0.832–1.200)

*p < 0.05, **p < 0.01.

**TABLE 3:** Heterogeneity tests for causal relationships of IDD with BPI and CTSG.

Exposure	Outcome	Method	Q	Q_df	Q_pval
BPI	IDD	MR Egger	5.692	9	0.770
		Inverse variance weighted	6.908	10	0.734
CTSG		MR Egger	21.015	22	0.520
		Inverse variance weighted	21.025	23	0.580

**TABLE 4:** Horizontal pleiotropy tests of IDD with BPI and CTSG.

Exposure	Outcome	Egger_intercept	SE	Pvalue
BPI	IDD	−0.008	0.007	0.299
CTSG	IDD	0.003	0.004	0.920

### 3.6 Pathways related to BPI and CTSG in IDD

**FIGURE 6:**
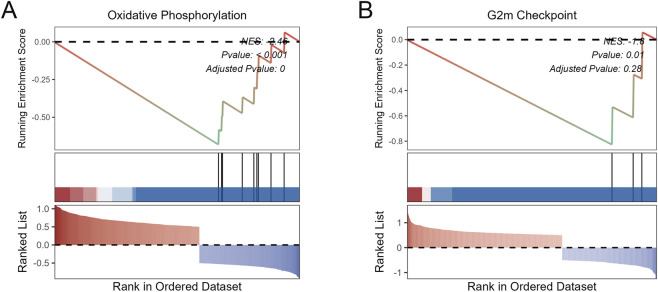
Pathways related to BPI and CTSG in IDD. **(A)** Pathway related to BPI. **(B)** Pathway related to CTSG.

To explore the potential mechanisms of BPI and CTSG in IDD, we performed GSEA. The results indicated that BPI was significantly negatively correlated with oxidative phosphorylation (Figure 6A), while CTSG was negatively correlated with the G2M checkpoint (Figure 6B).

### 3.7 Experimental validation

**FIGURE 7:**
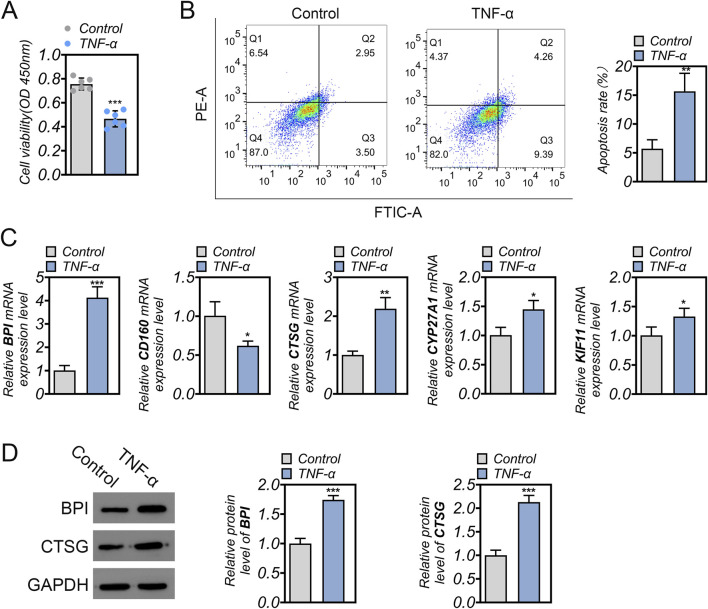
Expression validation of hub genes *in vitro*. **(A)** Cell viability was detected using CCK-8 analysis. **(B)** Cell apoptosis was detected using flow cytometry assay. **(C)** mRNA expression of BPI, CD160, CTSG, CYP27A1, and KIF11. **(D)** Protein expression of BPI and CTSG. Each experiment was performed in triplicate to ensure reproducibility. *p < 0.05, **p < 0.01, ***p < 0.001.

Finally, we validated the expression of the model genes through cellular experiments. TNF-α-induced NPCs were used to simulate IDD-related damage. Compared to the control group, cell viability was significantly reduced in the TNF-α group (p < 0.001, Figure 7A), while the apoptosis rate was markedly increased (p < 0.01, Figure 7B), indicating significant cellular damage in the TNF-α-treated NPCs. Consistent with the bioinformatics analysis, the damaged NPCs exhibited upregulated expression of BPI, CTSG, CYP27A1, and KIF11, while CD160 expression was downregulated (p < 0.05, Figure 7C). Western blotting was performed to further validate the protein expression of the two core genes, BPI and CTSG. As shown in Figure 7D, the protein levels of BPI and CTSG were significantly upregulated in the damaged NPCs compared to the control group (p < 0.001, Figure 7D).

**FIGURE 8:**
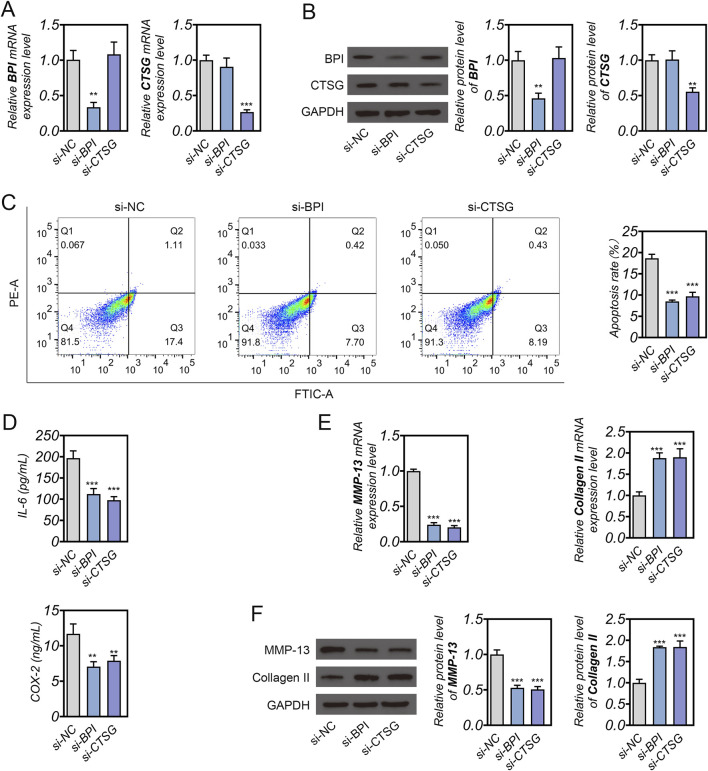
Functional validation of BPI and CTSG *in vitro*. **(A)** Quantitative real-time PCR analysis showing the efficiency of BPI and CTSG silencing in NPCs. **(B)** Western blot analysis and quantification of BPI and CTSG protein expression after siRNA transfection. **(C)** Flow cytometric analysis of apoptosis in NPCs transfected with si-NC, si-BPI, or si-CTSG. **(D)** ELISA results showing the secretion levels of inflammatory cytokines IL-6 and COX-2 in each group. **(E)** Relative mRNA expression levels of extracellular matrix-related genes MMP-13 and Collagen II. **(F)** Western blot analysis and quantification of MMP-13 and Collagen II protein levels. **p < 0.05, **p < 0.01, ***p < 0.001.

Furthermore, to explore the biological functions of BPI and CTSG in NPCs, gene silencing experiments were performed using siRNA. The knockdown efficiency was confirmed by qRT-PCR and Western blotting, which demonstrated that both mRNA and protein levels of BPI and CTSG were markedly reduced after siRNA transfection (p < 0.01, Figures 8A,B). Flow cytometric analysis revealed that the apoptotic rate of NPCs was significantly decreased following the knockdown of BPI or CTSG compared with the si-NC group (p < 0.001, Figure 8C), suggesting that silencing these genes could alleviate TNF-α-induced apoptosis. Moreover, the secretion of pro-inflammatory cytokines IL-6 and COX-2 was notably decreased in the si-BPI and si-CTSG groups (p < 0.01, Figure 8D), indicating a reduction in inflammatory response. To further investigate the effects of BPI and CTSG silencing on extracellular matrix metabolism, we assessed the expression of MMP-13 and Collagen II. qRT-PCR and Western blotting analyses revealed that inhibition of BPI or CTSG significantly suppressed MMP-13 expression while enhancing Collagen II levels (p < 0.001, Figures 8E,F). These findings indicate that silencing BPI and CTSG mitigates inflammation, inhibits matrix degradation, and promotes ECM synthesis in NPCs, thereby exerting a protective effect against IDD.

## 4 Discussion

This study conducted a comprehensive analysis of multiple GEO datasets to identify differentially expressed PDGs in IDD and explore their potential roles in disease onset and progression. By intersecting the DEGs in IDD with 4,463 PDGs, 14 differentially expressed PDGs in IDD were identified. Functional enrichment analysis indicated that these genes are primarily involved in immune defense responses, cell killing, neutrophil-related functions, and microbial infection pathways. Inflammation is closely associated with the occurrence and development of IDD, which can be categorized into septic and aseptic inflammation (Yang et al., 2024). Since IDD was first reported, the combined bacterial infection rate of intervertebral discs has been 25.3% over the past decades (Tang et al., 2021). The diffusion of inflammatory cytokines and chemokines from the intervertebral disc to surrounding tissues leads to increased local inflammation, further accelerating the degenerative process (Bermudez-Lekerika et al., 2022). The correlation between the 14 identified PDGs and inflammation- and infection-related pathways suggests that these genes may play a crucial role in the development and progression of IDD.

Immune cell infiltration analysis further revealed the critical role of neutrophils in IDD and identified a significant correlation between neutrophils and the 14 differentially expressed PDGs. Consistent with previous studies reporting neutrophil infiltration in IDD (Shamji et al., 2010), our CIBERSORT analysis showed that neutrophil infiltration levels were significantly elevated in IDD patients, while other immune cells did not exhibit significant differences. To validate the stability of our results, we employed two additional immune infiltration analysis algorithms, both of which yielded the same conclusions, further enhancing the reliability of our findings. A recent study has indicated that high neutrophil counts are an independent risk factor for IDD (Guo et al., 2024). Neutrophil infiltration in the intervertebral disc tissues of IDD patients may induce NPC pyroptosis through mechanisms such as the release of inflammatory mediators and the generation of reactive oxygen species (ROS), thereby exacerbating IDD progression (Shao et al., 2024; Guo et al., 2023). Correlation analysis further revealed that neutrophils were significantly negatively correlated with KIF11, FCGBP, and CD160, while positively correlated with FCGR1A, CYP4F2, CTSG, and BPI, suggesting that neutrophils may play a role in IDD by regulating specific genes.

Furthermore, this study explored the clinical value of the 14 PDGs. In constructing the diagnostic model, we used Lasso regression to identify five key genes—BPI, CD160, CTSG, CYP27A1, and KIF11—and developed an IDD diagnostic model based on these genes. This model demonstrated high diagnostic performance in both the training and test sets, with AUC values of 0.969 and 0.889, respectively, indicating strong discriminative ability for IDD. Notably, the test dataset used in this study consisted of blood samples, which holds significant clinical relevance. Compared to obtaining intervertebral disc tissue samples, blood sample collection is more convenient and non-invasive, making it suitable for large-scale clinical screening and early diagnosis (Boukovala et al., 2024).

To further investigate the causal relationship between the model genes and IDD, we conducted MR analysis. The results showed that among the five MR methods used, only BPI and CTSG exhibited a significant causal relationship with IDD. BPI is a multifunctional cationic protein produced by neutrophils in response to inflammatory stress (Yu et al., 2024). It has been shown to be downregulated in in vivo models of arthritis (Scanu Oliviero et al., 2022). CTSG is primarily expressed by neutrophils, B cells, and other immune cells, exhibiting both pro-inflammatory and anti-inflammatory activities (Yakupu et al., 2023). In wild-type mice with CTSC knockdown or treated with its inhibitor, impaired inflammatory gene expression was observed (Raith et al., 2024). Another study reported that CTSG in neutrophil extracellular traps contributes to pathogen elimination (Zhou et al., 2022). In this study, SNP effect plot analysis indicated a negative association between these two genes and IDD. Additionally, Cochran’s Q test and MR-Egger intercept analysis revealed no significant heterogeneity or horizontal pleiotropy in the causal relationship between IDD and BPI or CTSG, further reinforcing the reliability of the results. Single-SNP exclusion analysis also demonstrated that the effect direction of BPI and CTSG remained unchanged after removing any single SNP, indicating strong robustness of the findings.

Although MR analysis revealed a negative association between BPI and CTSG with IDD, interestingly, when we simulated the IDD-related injury environment by stimulating NPCs with TNF-α, we observed a significant upregulation of both BPI and CTSG at the gene and protein levels. Analysis based on public datasets also showed that these two genes were significantly upregulated in IDD. This apparent inconsistency may stem from the difference between genetically predicted expression and disease-induced expression. MR analysis reflects the impact of genetically determined, long-term, and constitutive expression levels on IDD risk, rather than transient regulation under inflammatory conditions. Genetically determined low expression of BPI/CTSG may confer protection against IDD onset, whereas their secondary upregulation during tissue injury may represent a compensatory inflammatory response. Once IDD occurs or the inflammatory environment is induced, BPI and CTSG may be upregulated as stress-responsive genes to modulate immune activity. Supporting this hypothesis, our functional experiments showed that knockdown of BPI or CTSG in TNF-α–stimulated NPCs significantly reduced apoptosis, suppressed the secretion of IL-6 and COX-2, downregulated MMP-13 expression, and increased Collagen II expression. These findings suggest that excessive expression of BPI/CTSG under inflammatory stress may exacerbate cell injury and extracellular matrix degradation. Moreover, correlation analysis revealed a significant positive association between BPI/CTSG and neutrophil infiltration. Therefore, we speculate that under chronic low-grade inflammation, upregulated BPI and CTSG may promote IDD progression by enhancing oxidative stress and activating MMP pathways (Liu et al., 2019; Wang Wang et al., 2024).

GSEA enrichment analysis further elucidated the potential mechanisms of BPI and CTSG in IDD. BPI was significantly negatively correlated with the oxidative phosphorylation pathway, whereas CTSG was significantly negatively correlated with the G2M checkpoint. Previous studies have demonstrated that mitochondrial dysfunction plays a critical role in the development and progression of IDD (Song et al., 2023; Kang et al., 2020). BPI is a cationic antimicrobial protein capable of regulating mitochondrial membrane potential and ROS generation in immune cells (Soto-Dávila and ChakrabortyRiseSantander, 2019; Iizasa et al., 2016; Xingyuan and Chen, 2020). We speculate that excessive expression of BPI in NPCs may indirectly inhibit oxidative phosphorylation by disrupting mitochondrial homeostasis, thereby leading to energy metabolism imbalance and increased oxidative stress, which aggravate IDD progression. Meanwhile, as a serine protease, CTSG has been identified as a gene associated with bone marrow differentiation (Dunne et al., 2006). Previous studies have shown that CTSG participates in the regulation of cell proliferation and extracellular matrix remodeling (Chan et al., 2023; Hua et al., 2025; Dai et al., 2024). Therefore, aberrant activation of CTSG may interfere with normal cell cycle regulation, leading to cell cycle arrest or senescence in NPCs and thereby accelerating the development of IDD.

Despite the robustness of our findings, this study has several limitations. First, although we analyzed multiple datasets and validated the results through independent cohorts, MR analysis, and *in vitro* experiments, validation in blinded clinical patient samples has not yet been performed. This limitation to some extent restricts the clinical translational potential of our findings. Future studies should collect and analyze large clinical specimens to further verify the diagnostic performance and clinical relevance of the five-gene model. Second, this study used TNF-α stimulation of NPCs to simulate the IDD environment, but the onset and progression of disc degeneration involve multiple factors, necessitating further validation of our findings in an *in vivo* setting. Furthermore, the downstream signaling pathways and cellular processes of BPI and CTSG implicated by bioinformatics analyses warrant further experimental validation in future studies.

## 5 Conclusion

In summary, this study integrated bioinformatics analysis, MR research, and experimental validation to reveal the critical roles of PDGs, especially BPI and CTSG, in IDD, further supporting the importance of neutrophils in the progression of IDD. Notably, the diagnostic model constructed based on blood samples offered the potential for non-invasive diagnosis of IDD and provided a more practical strategy for clinical detection and early screening. These findings not only offer new insights into the pathogenesis of IDD but also identify potential targets for its clinical diagnosis and treatment. Future studies should further explore the specific regulatory mechanisms of these genes to provide stronger evidence for the precise treatment of IDD.

## Data Availability

The datasets generated during and/or analysed during the current study are available from the corresponding author on reasonable request.
