# Supplementary material for: Identifying druggable gene-related biomarkers in intervertebral disc degeneration through transcriptome sequencing and mendelian randomization analysis
Source: Front Genet. 2026 Jan 23;17:1627091. doi: 10.3389/fgene.2026.1627091 (PMC12875596; doi:10.3389/fgene.2026.1627091)
Supplement: Supplementary file 1 [file Table1.docx]

**Table S1 Summary of GWAS datasets used for MR analysis**

| Variables | | IEU GWAS ID | Population | Sample size |
| --- | --- | --- | --- | --- |
| Outcome | IDD | finn-b-M13_INTERVERTEB | European | NA |
| Exposure | BPI | eqtl-a-ENSG00000101425 | European | 31684 |
|  | CD160 | eqtl-a-ENSG00000117281 | European | 14263 |
|  | CTSG | eqtl-a-ENSG00000100448 | European | 31684 |
|  | CYP27A1 | eqtl-a-ENSG00000135929 | European | 31684 |
|  | KIF11 | eqtl-a-ENSG00000138160 | European | 31470 |
